# Alterations of sirtuins in mitochondrial cytochrome c-oxidase deficiency

**DOI:** 10.1371/journal.pone.0186517

**Published:** 2017-10-23

**Authors:** Arne Björn Potthast, Theresa Heuer, Simone Johanna Warneke, Anibh Martin Das

**Affiliations:** Clinic for Paediatric Kidney-, Liver-, and Metabolic Diseases, Hannover Medical School, Hannover, Germany; Universidad Pablo de Olavide, SPAIN

## Abstract

**Background:**

Sirtuins are NAD^+^ dependent deacetylases, which regulate mitochondrial energy metabolism as well as cellular response to stress. The NAD/NADH-system plays a crucial role in oxidative phosphorylation linking sirtuins and the mitochondrial respiratory chain. Furthermore, sirtuins are able to directly deacetylate and activate different complexes of the respiratory chain. This prompted us to analyse sirtuin levels in skin fibroblasts from patients with cytochrome c-oxidase (COX) deficiency and to test the impact of different pharmaceutical activators of sirtuins (SRT1720, paeonol) to modulate sirtuins and possibly respiratory chain enzymes in patient cells in vitro.

**Methods:**

We assayed intracellular levels of sirtuin 1 and the mitochondrial sirtuins SIRT3 and SIRT4 in human fibroblasts from patients with COX- deficiency. Furthermore, sirtuins were measured after inhibiting complex IV in healthy control fibroblasts by cyanide and after incubation with activators SRT1720 and paeonol. To determine the effect of sirtuin inhibition at the cellular level we measured total cellular acetylation (control and patient cells, with and without treatment) by Western blot.

**Results:**

We observed a significant decrease in cellular levels of all three sirtuins at the activity, protein and transcriptional level (by 15% to 50%) in COX-deficient cells. Additionally, the intracellular concentration of NAD^+^ was reduced in patient cells. We mimicked the biochemical phenotype of COX- deficiency by incubating healthy fibroblasts with cyanide and observed reduced sirtuin levels. A pharmacological activation of sirtuins resulted in normalized sirtuin levels in patient cells. Hyper acetylation was also reversible after treatment with sirtuin activators. Pharmacological modulation of sirtuins resulted in altered respiratory chain complex activities.

**Conclusions:**

We found inhibition of situins 1, 3 and 4 at activity, protein and transcriptional levels in fibroblasts from patient with COX-deficiency. Pharmacological activators were able to restore reduced sirtuin levels and thereby modulate respiratory chain activities.

## Introduction

Mitochondriopathies (mitochondrial respiratory chain defects) are severe, often life-threatening inborn errors of energy metabolism. Only symptomatic treatment is available for these multisystemic diseases which can affect almost any organ system.

The mitochondrial respiratory chain is responsible for the bulk of energy production in humans. It consists of four complexes, which transfer electrons from NADH (nicotinamide adenine dinucleotide, reduced form) and FADH_2_ (flavin adenine dinucleotide, reduced form) to oxygen as terminal electron acceptor, thus producing H_2_O. During this process, the complexes generate a proton gradient across the inner mitochondrial membrane [1]. Complex V or ATP-synthase can use this electrochemical gradient to synthesize adenosine triphosphate (ATP). In oxidative phosphorylation electrons are transferred to oxygen molecules thus producing radical oxygen species (ROS) like superoxide (O2-). ROS are cellular stressors which may lead to damage of DNA, proteins or lipid membranes.

In inborn errors of the respiratory chain (RC) reduced activities of these complexes result in compromised energy supply leading to cellular energy deficiency and dysfunction, especially in organs with high energy demand like muscle, heart and brain. The energy flux may vary considerably, for example by a factor of 5–10 in heart muscle [2]. Apart from passive regulation of ATP production via substrate (ADP) saturation, various active regulatory elements are operational like the mitochondrial ATPase inhibitor protein IF_1_ [3,4], the Ca^2+^- binding inhibitor protein [5], nitric oxide (NO) [6] and SIRT1 and SIRT3 [7].

Sirtuins are NAD^+^ (nicotinamide adenine dinucleotide, oxidized form) dependent enzymes, which are evolutionary highly conserved in prokaryotes and eukaryotes [8]. The silent information regulator 2 (Sir2) was initially discovered in *Saccharomyces cerevisiae* and was described as a histone deacetylase class III. In mammals, seven different sirtuins (SIRT1-SIRT7) [9] localised in different cell compartments have been described [10]: i) nucleus (SIRT1, SIRT6 and SIRT7), ii) cytosol (SIRT1 and SIRT2) and iii) mitochondria (SIRT3, SIRT4 and SIRT5).

Structurally, all sirtuins share a NAD^+^-binding domain, where the essential cofactor NAD^+^ is bound to the enzyme and a catalytic domain for the specific enzymatic reaction. The most common enzymatic reaction of sirtuins is a deacetylase function [11] present in all sirtuins. Additionally, further deacylation reactions catalysed by sirtuins are demalonylation, desuccinylation and deglutharylation for SIRT5 [12] or long chain fatty acyl groups as described for PfSir2A [13]. Furthermore SIRT4 and SIRT6 have an ADP-ribosylation activity [14,15].

Sirtuins are linked to a wide range of cellular processes: stress response [16], metabolic regulation [17–20], aging [21,22] and cancer [23]. Recent studies showed an over-all acetylation level of 65% of the mitochondrial proteome [24]. Based on these findings, SIRT3 may be the main regulator of mitochondrial activity at the level of RC [17], SIRT3 deacetylates NADH dehydrogenase (ubiquinone) 1 alpha subcomplex 9 (NDUFA9), a subunit of complex I [24], succinate dehydrogenase complex, subunit A (SDHA), subunit of complex II [25], and several subunits of complex V [26]. Deacetylation leads to an activation of RC-complexes. Recent studies showed an additional regulation of the RC by SIRT5. SIRT5 desuccinylates different subunits of complex I (NDUFA7, NDUFA12, NDUFA) and complex II (DHSA) [27].

SIRT3 is also involved in the cellular response to oxidative stress. It deacetylates the existing pool of mitochondrial manganese superoxide dismutase (SOD2) [28,29] thus inducing SOD2 activity. SOD2 transcription is regulated by FOXO3, which is deacetylated and thereby activated by SIRT3, leading to an increased transcription rate [30]. Furthermore, SIRT3 affects ROS detoxification by inducing the glutathione system as well as the thioredoxin system [31].

The function of SIRT4 is less well studied. It is linked to insulin secretion during caloric restriction [14] and plays a role in regulating long chain fatty acid oxidation at the level of malonylCoA [32]. Recent studies suggest an important role of SIRT4 in ATP homeostasis by regulating the adenine nucleotide translocator 2 (ANT2) and therefore oxidative phosphorylation [33]. Additionally, a role for SIRT4 in response to genotoxic stress [34] and tumour suppression [35] is discussed in the context of SIRT4 as a regulator of mitochondrial glutamine metabolism.

SIRT1 and SIRT3 are involved in the regulation of mitochondrial biogenesis via a AMPK–SIRT1–PGC1α pathway [36,37]. AMP activated protein kinase (AMPK) is an important regulator of cellular energy metabolism. It phosphorylates SIRT1 leading to an increased enzyme activity of SIRT1, which deacetylates peroxisome proliferator activated receptor coactivator 1α (PGC1α) [38,39], resulting in altered transcription of metabolic genes. Due to the fact that AMPK and SIRTs are dependent on cofactors from energy metabolism they act as sensors of metabolic state of the cell and can act as a fast response to energy shortage.

Recently, pharmacological activators of different sirtuins were discovered [9]. Resveratrol and SRT1720 are the most common pharmaceuticals for SIRT activation [40]. Both compounds are effective SIRT1 activators but lack a sufficient activation of other sirtuins in vitro. Additionally, it has been demonstrated that SRT1720 induced mitochondrial biogenesis under caloric restriction as well as after acute oxygen injury [41].

The novel sirtuin-activator paeonol (2’-Hydroxy-4’-methoxyacetophenone) was recently discovered [42]. Paeonol prevented premature senescence induced by oxidative stress in HUVECs by modulating the expression of SIRT1 [42] and is able to induce cortical cytochrome oxidase [43].

We have previously shown secondary down-regulation of ATPsynthase in RC defects which presumably serves energy conservation [44]. If RC function were compromised in mitochondriopathies, NADH accumulates and may result in reduced levels of the sirtuin cofactor NAD^+^ which could affect sirtuin function. We studied sirtuin function in fibroblasts from children with clinical and biochemical features of COX-deficiency which is supposed to be the most severe defect at the final step of the RC.

## Material and methods

### Ethics statement

Human skin fibroblasts of patient cells were primarily obtained for clinical diagnostic purposes. All patients agreed to the use of fibroblasts for scientific purposes. All fibroblasts of healthy donors were obtained during routine surgeries and donors gave informed consent for the scientific use of the cells. We received a positive vote from the Ethics Committee at Hannover Medical School prior to the study (EC-vote Nr. 5176)

### Fibroblasts and cell cultures

Human skin fibroblasts obtained with informed consent from healthy individuals undergoing routine surgery were used as controls. Fibroblasts from six patients with clinical symptoms of RC-deficiency and reduced activities of complex IV were used for experimentation and compared to age-matched healthy controls (passages 4 to 9). Clinical and biochemical details of the patients are given in Table 1.

**Table 1:** Complex IV activity in nmol / min per mg protein in fibroblasts and clinical symptoms of patients.

Patients	Complex IV Activity	Clinical symptoms	Died
Pat. 1	0	psychomotor retardation, cardiomyopathy, muscular hypotonia, epilepsy	No
Pat. 2	0	psychomotor retardation, epilepsy	yes
Pat. 3	0	psychomotor retardation, epilepsy	yes
Pat. 4	17	psychomotor retardation, muscular hypotension, Fanconi syndrome	yes
Pat. 5	0	psychomotor retardation, cardiomyopathy, epilepsy	yes
Pat. 6	10	psychomotor retardation, nephrotic syndrome, muscular hypotonia	no

(healthy children range): 27–92

The cells were cultured in 75-cm^2^ polystyrene flasks (Sarstedt AG, DE) using Dulbecco´s Modified Eagles Medium (DMEM) (Life Technologies GmbH., DE), containing 10% (v/v) heat inactivated fetal bovine serum (Biowest SAS, FR) and 1% (v/v) penicillin/ streptomycin (PAA Laboratories GmbH, DE). The cells were incubated at 37°C and 5% CO_2_.

All experiments included measurements in 4 different fibroblast cell lines and were repeated independently in triplicate. Prior to analysis, cells were lysed using RLT buffer (Qiagen, DE) for RNA isolation, Laemmli buffer for Western Blot and Hepes buffer for enzymatic assays.

### NaCN treatment of cell

We mimicked the effect of COX-deficiency by incubating healthy fibroblasts with an increasing concentration of sodium cyanide (NaCN). The cells were grown to 70% confluency, then cells were incubated at concentrations of 0 mM (control), 0.75 mM, 2.5 mM, 5 mM or 7.5 mM of NaCN for 5 days.

Efficacy of COX-inhibition by cyanide was judged by measuring oxygen consumption using an XF Analyser (Seahorse Bioscience). Toxicity of cyanide treatment was assessed by measuring LDH-release from the cells [45]. After 3 days we changed the media to renew nutrients and NaCN. The cells were lysed for further analysis after five days of incubation.

### Treatment of cells with SIRT modulators

In an attempt to rescue the SIRT enzyme function in COX- deficient cells we treated cultured fibroblasts with different pharmacological substances.

Fibroblasts of control and patient cells were cultured until they were 60% confluent. Subsequently, they were either treated with 10 μM SRT1720, 50μM paeonol, 350μM suramin, 10 μM tenovin-6 or DMSO as a vehicle control or remained untreated as control. The fibroblasts were treated for 7 days with media changes after two and five days to renew nutrients and pharmacological substances.

### RNA isolation and qRT-PCR

RNA was isolated with RNeasy Mini Kit (Qiagen, DE) according to the product protocol. The isolated RNA was reverse transcribed to cDNA with Omniscript RT Kit (Qiagen, DE). Real-time PCR of different cDNA samples was carried out with SYBR green on a 7900 HT fast real-time PCR system (Applied Biosystems, DE). The following Primers were used for the analysis:

*human β-actin (forward)* TTC CTG GGC ATG GAG TC; *human β-actin (reverse)*: CAG GTC TTT GCG GAT GTC; *human GAPDH (forward)*: ACG TGT CAG TGG TGG ACC TG; *human GAPDH (reverse)*: AGT GGG TGT CGT GTT GAA GT; *human HPRT1 (forward)*: GCT GAC CTG CTG GAT TAC; *human HPRT1 (reverse)*: TGC GAC CTT GAC CAT CTT; *human SIRT1 (forward)*: CAA CTT GTA CGA CGA AGA C; *human SIRT1 (reverse)*: TCA TCA CCG AAC AGA AGG; *human SIRT3 (forward)*: CAG TCT GCC AAA GAC CCT TC; *human SIRT3 (reverse)*: AAA TCA ACC ACA TGC AGC AA; *human SIRT4 (forward)*: GCT GTG AGA GAA TGA AGA TGA GC; *human SIRT4 (reverse)*: CTT GGA AAG GGT GAT GAA GCG. β-actin, GAPDH and HPRT1 were used as loading controls. Relative changes in the mRNA expression were calculated according to Vandesompele et al. [46]

### Protein isolation and Western blotting

Cells (5000 cells/μl) were lysed in 3x Laemmli-buffer. To disrupt all cells, the suspension was heated for 10 min at 95°C followed by centrifugation at 8,000 x g.

10 μl (~20 μg) of the cell lysate were loaded on 10%-SDS-PAGE and separated. Transfer to a nitrocellulose membrane was performed with semi-dry blot (Biometra, DE) at 200 mA for 20 min. We used primary antibodies for SIRT1 (Merck Millipore, USA), SIRT3 (Merck Millipore, USA), SIRT4 (Thermo Scientific, DE), AMPKα1 (Merck Millipore, USA), PPARγ (Merck Millipore, USA), PGC1α (Merck Millipore, USA) and β-Actin (Cell Signalling, DE) as a marker. For detection, we used the Odyssey FC system (LI-COR Bioscience, USA) with appropriate secondary antibodies.

### Protein isolation and Western blotting for acetylated proteins

Cells were lysed in RIPA-buffer (50 mM Tris-HCl, 1% (v/v) NP-40, 0.25% (w/v) sodium deoxycholate, 150 mM NaCl, 1 mM EDTA, 1 mM PMSF, 1 mM sodium fluorite, pH 7.4) using a cell scraper followed by sonification for 10 seconds with 20 kHz and an amplitude of 75%. Protein concentration was measured using the BCA Protein Assay Kit (Thermo Scientific, US).

50 μg total protein were loaded on 10%-SDS-PAGE and separated. Transfer to a nitrocellulose membrane was performed with a semi-dry blot (Biometra, DE) at 200 mA for 20 min. To detect the total acetylation level of the cells we used an anti-acetylated protein antibody (Abcam, GB) and β-Actin (Cell Signalling, DE) as loading control. For detection, we used the Odyssey FC system (LI-COR Bioscience, USA) with appropriate secondary antibodies.

### Sirtuin deacetylase activity assay

SIRT1 and SIRT3 deacetylase activities were determined by using a SIRT1/SIRT3 fluorometric drug discovery assay kit (Enzo Life Science, CH). To ensure that we measure maximal activities we added a surplus of NAD^+^ to our assays. We followed the manufacturer’s protocol with 7500 lysed cells in HEPES buffer (110 mM NaCl, 2.6 mM KCl, 1.2 mM KH2PO_4_, 1.2 mM MgSO4x7H2O, 1.0 mM CaCl2, 25 mM HEPES).

### Respiratory chain activity

The enzymatic activities of complex I-IV and the ATP-Synthase (Complex V) of the mitochondrial respiratory chain were measured as described in earlier publications.[47] Complexes I+III, II+III and Complex IV were determined spectrophotometrically using rotenone and antimycin A as specific inhibitors. ATP synthase activity was measured spectrophotometrically with oligomycin as an inhibitor.

### Intracellular NAD^+^ concentration

We took 250 μl of the Hepes-lysate used for the Sirtuin deacetylase activity assay precipitated the protein by adding 900 μl 0.5% (w/v) sodium deoxycholate and 100 μl of 50% (w/v) trichloroacetate (TCA) after 15 min the protein was spun-down by centrifugation for 10 min at 13,000 x g and 4°C.

The supernatant was used for NAD^+^ measurement according to Skokowa *et*. *al*. [48].

### Statistical analysis

**Fig 1:**
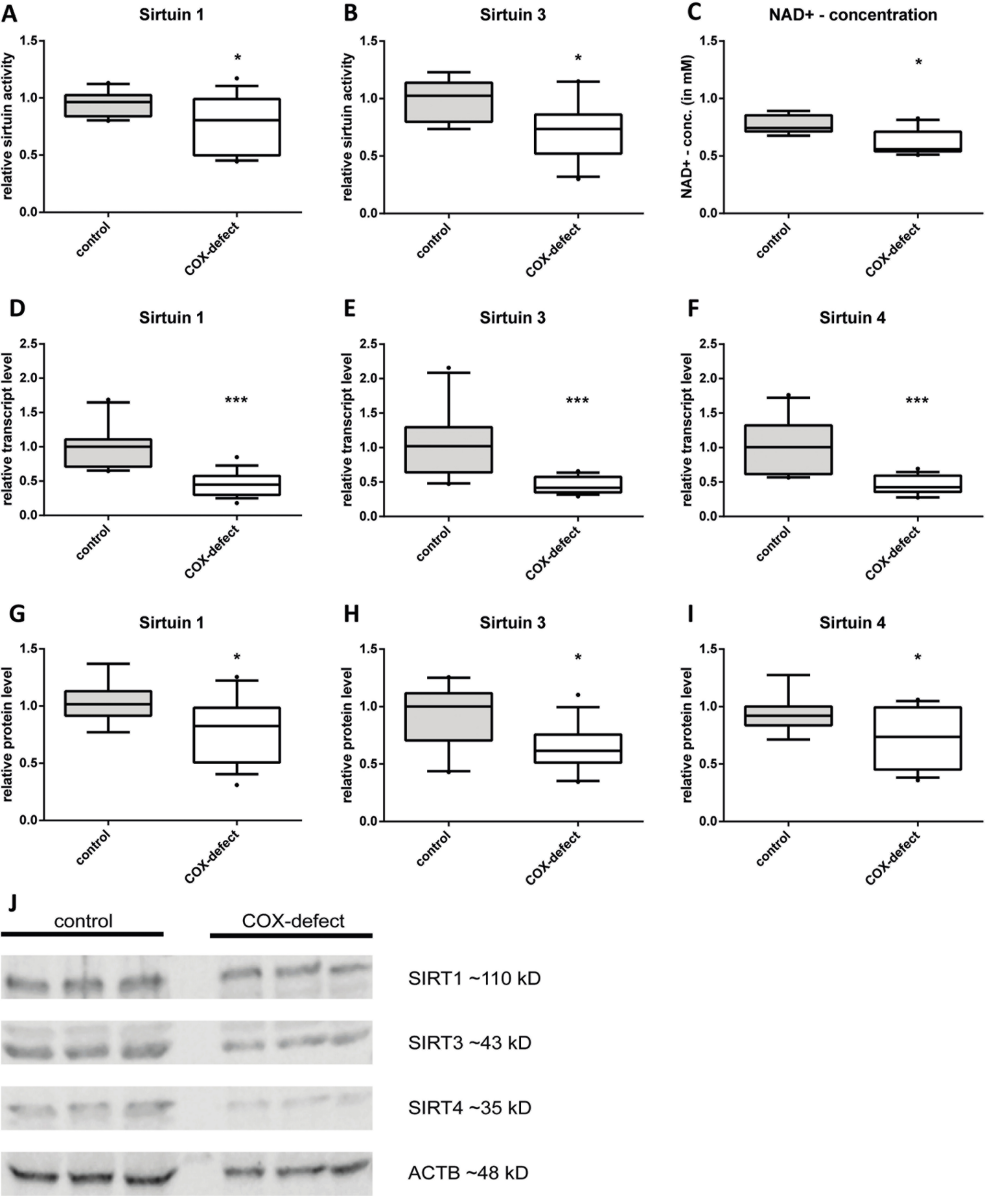
Complex IV deficiency led to decreased levels of SIRT1, SIRT3 and SIRT4 as well as decreased NAD^+^ concentrations. (A-B) Relative deacetylase activity of SIRT1 and SIRT3. (C) Intracellular NAD^+^ concentrations in healthy control fibroblasts and fibroblasts obtained from complex IV-deficient patients. (D-F) Transcript levels of *sirt1*, *sirt3* and *sirt4* (G-I) Relative protein levels of SIRT1, SIRT3 and SIRT4 in complex IV deficient fibroblasts compared to control cells. (J) Representative Western blot of COX-deficient cells compared to control cells. (n = 6; complex IV deficient fibroblasts vs control cells, median with 5% and 95% quantiles; * p < 0,05, ** p < 0,01, *** p < 0,001).

**Fig 2:**
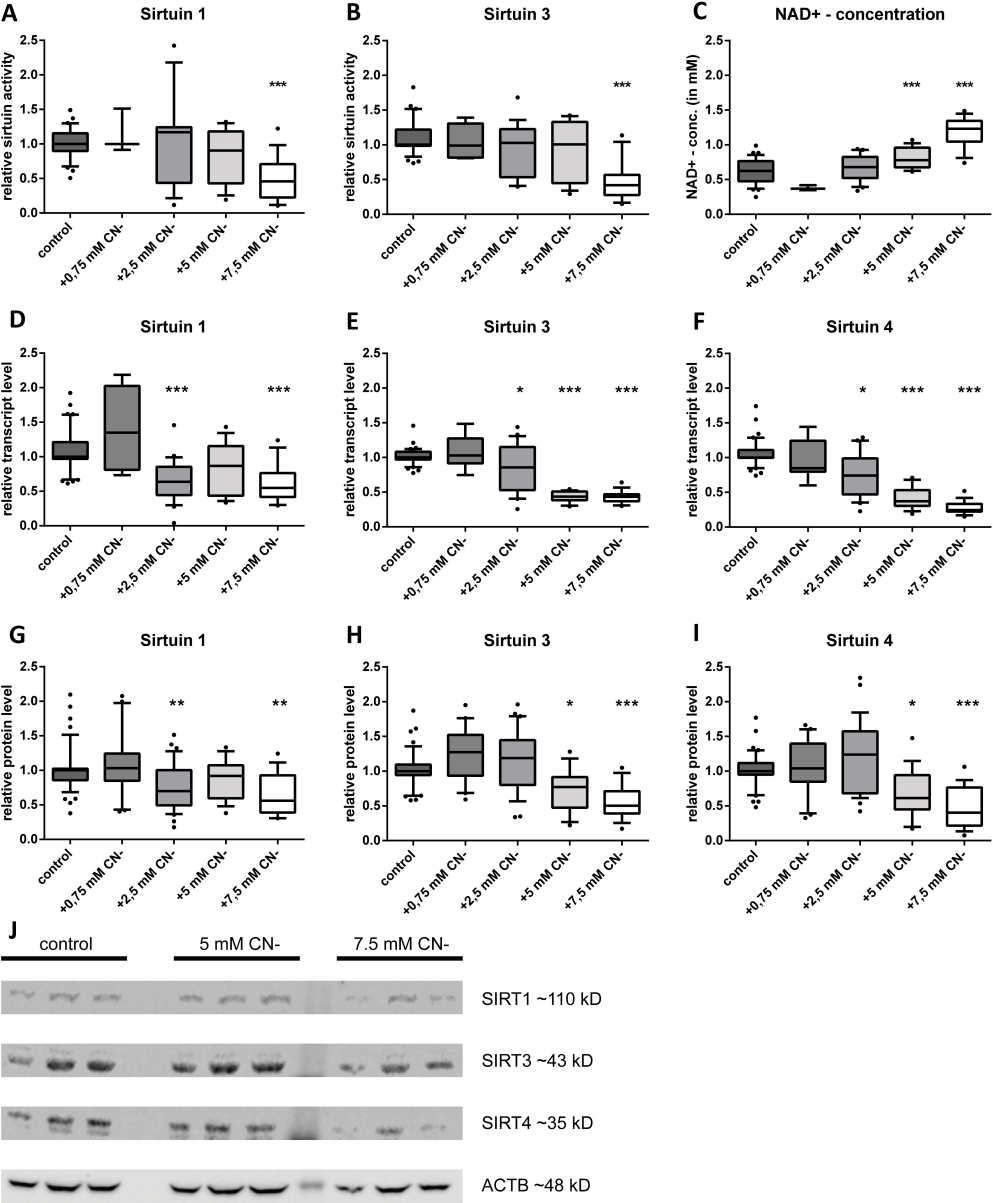
Sodium cyanide decreased intracellular SIRT1, SIRT3 and SIRT4 levels in a dose-dependent manner, whereas the intracellular NAD^+^-concentration was increased in a dose-dependent way. Relative deacetylase activity of SIRT1 (A) and SIRT3(B). Always shown as different NaCN concentrations compared to control cells. (C) Intracellular NAD^+^ concentrations in healthy control fibroblasts and fibroblasts treated with increasing sodium cyanide concentrations. (D-F) Transcript levels of *sirt1*, *sirt3* and *sirt4*. The mRNA levels are shown with increasing NaCN concentrations compared to the untreated control. (G-I) Relative protein levels of SIRT1, SIRT3 and SIRT4 in control cells compared to NaCN treated cells. (J) Representative Western blots of SIRT1, SIRT3 and SIRT4 as well as ACTB as internal control. (n = 5; NaCN treated fibroblasts vs control cells, median with 5% and 95% quantiles; * p < 0.05, ** p < 0.01, *** p < 0.001).

**Fig 3:**
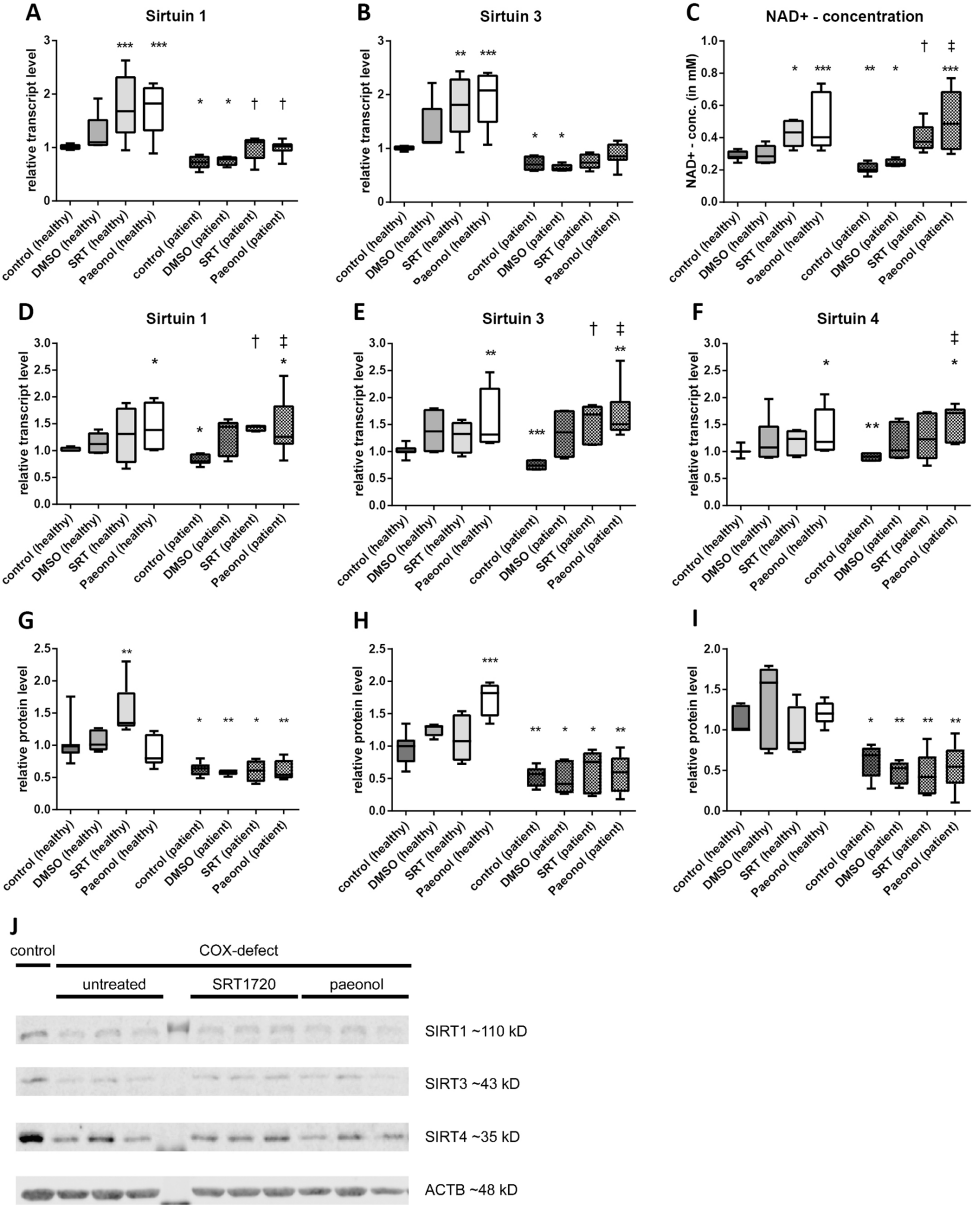
Activation of sirtuin levels with SRT1720 and paeonol rescued sirtuin levels in COX-deficiency. Relative deacetylase activity of SIRT1 (A) and SIRT3 (B) for treatment with DMSO (vehicle control), SRT1720 and paeonol in control cells and COX-deficient cells (C) Intracellular NAD^+^ concentrations in healthy control fibroblasts and COX-deficient fibroblasts treated with DMSO, SRT1720 and paeonol. (D-F) Transcript levels of *sirt1*, *sirt3* and *sirt4*. The mRNA levels are shown with DMSO (vehicle control), SRT1720 and paeonol treatment compared to the untreated controls. (G-I) Relative protein levels of SIRT1, SIRT3 and SIRT4 in control cells compared to fibroblasts treated with DMSO, SRT1720 and paeonol. (J) Representative Western blots of SIRT1, SIRT3 and SIRT4 as well as ACTB as internal control. (n = 5–10; Treatment with DMSO, SRT1720 and paeonol compared to untreated control cells, mean ± SD; * p < 0.05, ** p < 0.01, *** p < 0.001; Treatment with DMSO, SRT1720 and paeonol compared to untreated COX-deficient cells, median with 5% and 95% quantiles; †< 0.05, ‡ p < 0.01).

**Fig 4:**
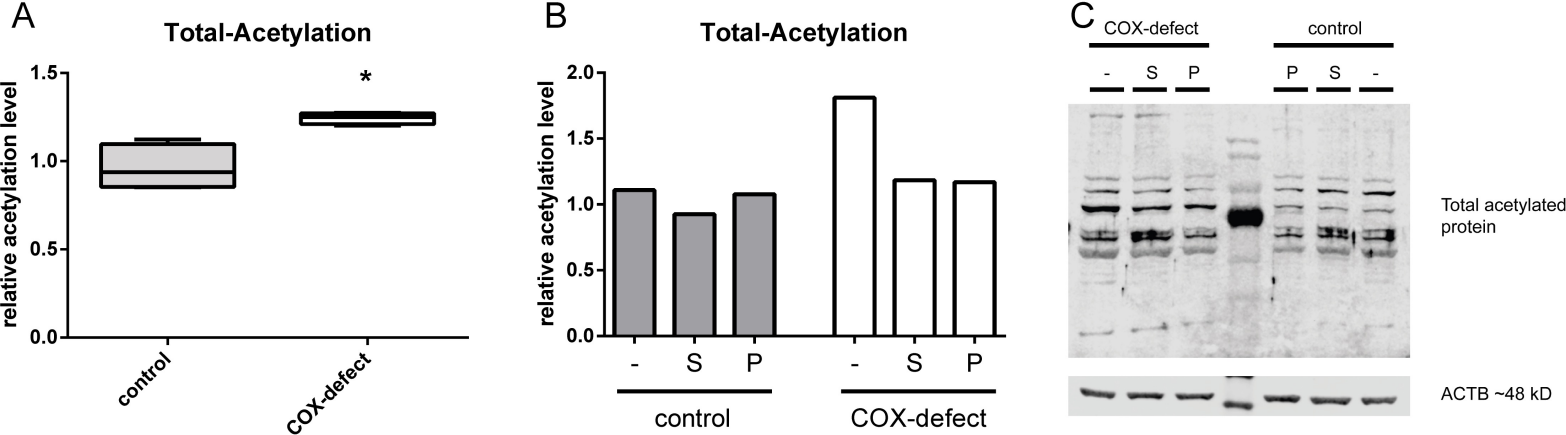
Total acetylation of COX-deficient cells with and without SRT1720 and paeonol treatment. (A) Total acetylation level of control fibroblasts compared to COX-deficient cells (B) Total acetylation level of COX-deficient cells treated with SRT1720 [S] and paeonol [P] compared to treated and untreated [–] control fibroblasts. (C) Representative Western blot of total acetylation of COX-deficient cells and control cells treated with SRT1720 [S], paeonol [P] or untreated [–]. n = 3; Treatment with SRT1720 and paeonol compared to untreated control cells, median with 5% and 95% quantiles; * p < 0,05, ** p < 0,01.

Results are shown as means ± standard deviation (SD). Data were parametrically distributed. To determine the statistical significance we used t-test (Fig 1), one-way ANOVA (Fig 2) and Kruskal-Wallis test (Figs 3 and 4). Measurements were either compared to the untreated or healthy controls (*) or in case of Fig 3 also compared to untreated patient cells (†). Differences were significant at p-values of 0.05 (*), 0,01 (**) and 0.001 (***).

For all statistical analyses we used GraphPad Prism version 6.01 (GraphPad Software Inc., USA).

## Results

### Analysis of sirtuin levels in COX-deficiency

As a functional parameter, we first analysed the active amount of the SIRT-proteins. In the absence of a reproducible assay for analysing SIRT4 activity, we focused on measuring SIRT1 and SIRT3 activity levels.

We found SIRT1 activity reduced by 15% (Fig 1 A), mean absolute activity of control cells was ~12 U/10,000 cells. The effect was more pronounced for SIRT3 activity, which showed a reduction by 25% (Fig 1 B) compared to control cells with an activity of ~36 U/10,000 cells.

As the sirtuin activity depends on the concentration of the cofactor NAD^+^, we determined the cellular NAD^+^ concentration. In patient cells the NAD^+^ levels were decreased by 21% (Fig 1 C) compared to control cells (0.77 mM).

At transcript level, *sirt3* and *sirt4* were decreased by 55% (Fig 1E and 1F), *sirt1* was reduced by 60% compared to control cells (Fig 1D). Reduced sirtuin expression in the patient cells resulted in reduced protein levels of the analyzed sirtuins. SIRT3 and SIRT4 were reduced by about 40% (Fig 1H and 1I). In contrast to the markedly decreased transcript level, SIRT1 was only reduced to 80% of controls at protein level (Fig 1G) suggesting a transcript or protein stabilisation. Taken together, our data showed a highly significant reduction of mitochondrial sirtuins as well as the nuclear SIRT1.

### NaCN treatment mimicked the effect of COX-deficiency

The oxygen consumption rate fell to 30% in the cells treated with 2.5 mM cyanide and was completely absent at 5–7.5 mM cyanide (supporting data S1 Fig). Whether LDH-activities were significantly increased in treated cells nor cellcounts were significantly reduced, both indicating cell viability (supporting data S8 Fig and S1 Table). The cell count was not significantly altered by incubation in the presence of cyanide.

The activity levels of SIRT1 and SIRT3 were reduced in response to NaCN treatment. Reduction was significant at concentrations of 5 mM and 7.5 mM. At 7.5 mM NaCN SIRT1 and SIRT3 activities were both reduced to 50% compared to control cells (Fig 2A and 2B).

While the NAD^+^ levels were reduced in COX-deficient cells, NaCN treatment led to an increase in a dose-dependent manner. Incubation of cells in the presence of 7.5 mM NaCN doubled the NAD^+^ concentration in the cells (Fig 2C).

At transcript level, we detected a reduction of sirtuins by cyanide in a dose-dependent manner. Starting at a concentration of 2.5 mM we could detect markedly reduced transcript levels of *sirt1*, *sirt3* and *sirt4* (Fig 2D–2F). Dose-dependency was most pronounced for *sirt4* (Fig 2F). The *sirt1* transcript was reduced by 40%, whereas transcripts of the mitochondrial sirtuins *sirt3* and *sirt4* were reduced by 60% and 80%, respectively.

The pattern observed at transcript level was reflected at protein level. We found significant reductions starting at 5 mM NaCN except for SIRT1, where we could observe a significant reduction of the protein amount already at 2.5 mM (0.69) (Fig 2G). SIRT3 was reduced to 55% (Fig 2H) whereas SIRT4 even dropped to 38% (Fig 2I)

### Pharmacological activation rescued decreased sirtuin levels

SRT1720 treatment resulted in an induction of SIRT1 activity to ~80% of control cells. In patient cells we could observe an induction by 40%, which was the activity level in control cells (Fig 3A). The effect of SRT1720 on SIRT3 activity was less pronounced. While there was a significant reduction of SIRT3 activity in patient cells, the treatment with SRT1720 was not able to rescue the SIRT3 activity to normal levels (Fig 3B). In patient cells SRT1720 treatment only resulted in a 10% induction of SIRT3 activity, while there was an 80% induction in healthy controls. Treatment with DMSO as vehicle control did not show any significant changes.

A similar effect was observed after treatment with paeonol; SIRT1 activity in controls was increased by 70% and by 40% in patient cells which showed a normal activity after paeonol treatment (Fig 3A). In contrast to SRT1720 we could detect an effect of paeonol on SIRT3 activity. In control cells, paeonol treatment almost doubled the activity (1.95) while it rescued SIRT3 activity to 88% of control cells (induction of 25% compared to untreated patient cells) (Fig 3B). Since paeonol like SRT1720 was dissolved in DMSO the vehicle control with DMSO served as a control group.

Additionally, we analyzed intracellular NAD^+^ concentration because it is the limiting cofactor for the sirtuin reaction. In patient cells we could detect a decreased NAD^+^ concentration (Fig 3C) (control cells = 0.29 mM; patient cells = 0.18 mM). Treatment with DMSO showed no changes in NAD^+^ concentration while both SRT1720 and paeonol treatment resulted in increased NAD^+^ concentrations. The effect was more pronounced with paenol (control cells = 0.49 mM; patient cells = 0.50 mM) than with SRT1720 treatment (control cells = 0.43 mM; patient cells = 0.40 mM) (Fig 3C).

Analyzing the transcript and protein levels after treatment with SRT1720 and paeonol showed comparable results. The patient cells which were analyzed in these experiments showed a reduction in *sirt1*, *sirt3* and *sirt4* transcript levels of 20% (*sirt1*), 25% (*sirt3*) and 20% (*sirt4*). After 7 days of SRT1720 treatment we observed an induction of *sirt1* mRNA by 27% in control cells and 39% in patient cells, respectively (Fig 3D). The mRNA expression was also increased after treatment with paeonol, where we found an induction at transcript levels by 41% in control cells and 38% in COX-deficient cells. For *sirt3* there was a significant induction of mRNA expression after treatment with paeonol in control (1.6) and patient cells (1.7) (Fig 3E), while SRT1720 showed a significant increase only in patient cells (1.5) whereas the effect of SRT1720 in control cells was comparable to treatment with DMSO (DMSO = 1.32; SRT1720 = 1.28). *sirt4* seems to be regulated just like *sirt3*. There is a small effect of the vehicle DMSO on *sirt4* mRNA expression although this effect is not significant (Fig 3F). Treatment with SRT1720 did not affect the transcription in control cells (1.16) while there was a not significant induction in patient cells (1.32). Paeonol in contrast, showed significant effects in patient cells (1.53) as well as in control cells (1.35).

Interestingly, the changes in mRNA expression of *sirt1*, *sirt3* and *sirt4* were not reflected at protein level. As described before, we measured significantly decreased protein levels of the three analyzed sirtuins; in these experiments SIRT1 showed a reduction to 63%, SIRT3 a reduction to 55% and SIRT4 a reduction to 61%. Treatment with SRT1720 and paeonol resulted only in little effects on protein levels. We could observe an induction of SIRT1 protein after SRT1720 treatment in control cells (Fig 3G) and an induction of SIRT3 protein after paeonol treatment (Fig 3H). There were no effects of the sirtuin activators on SIRT1, SIRT3 and SIRT4 protein levels in patient cells (Fig 3G–3I).

### Effect of sirtuin modulation on total acetylation level of cells

We found an increase in the total acetylation level of COX-deficient cells. There was a hyper acetylation by about 40% in patient cells (Fig 4A). Treatment with SRT1720 and paeonol decreased hyper acetylation (Fig 4B). SRT1720 treatment resulted in a hyper acetylation of 28% whereas we measured a hyper acetylation of 63% in untreated cells. The effect of paeonol was even more pronounced since there was a hyper acetylation of only 8%, which means normalization.

### Influence of sirtuin modulation on the mitochondrial respiratory chain

**Fig 5:**
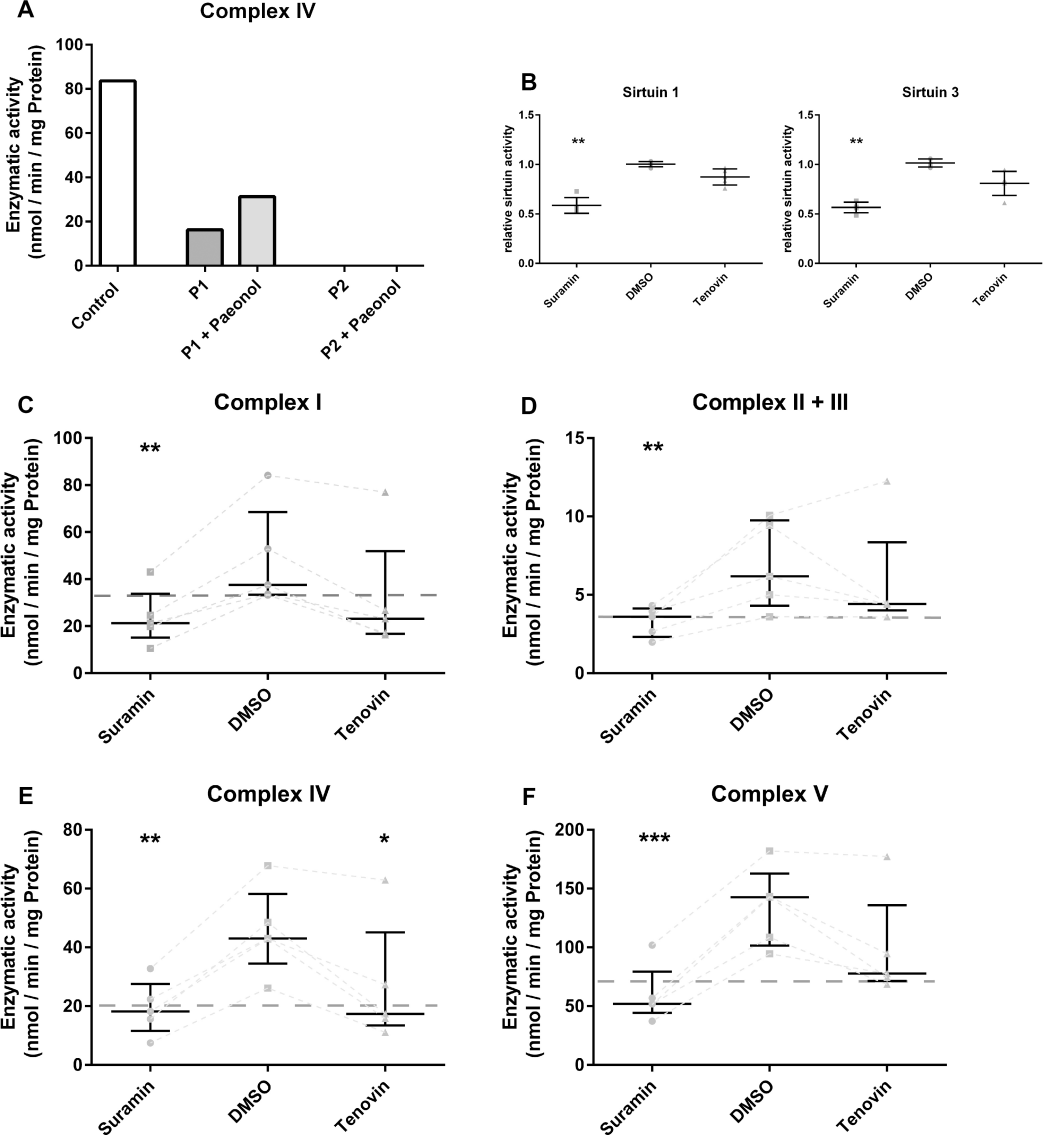
RC activity after pharmacological modulation of sirtuin activities. (A) Complex IV activity in untreated controls compared to patient cells with residual (P1) and without residual (P2) COX-activity. Both patient cell lines were treated with paeonol as sirtuin activator (B) Relative deacetylase activity of SIRT1 and SIRT3 after treatment with suramin and tenovin-6 compared to DMSO treated healthy fibroblasts. (C-F) Enzymatic activities of complex I (C), complex II+III (D), complex IV (E) and complex V (F) in DMSO treated control cells compared to cells treated with sirtuin inhibitors suramin and tenovin. (A: n = 1; B-F: n = 5; Treatment with paeonol, suramin and tenovin-6 compared to untreated control cells, median with 5% and 95% quantiles; * p < 0.05, ** p < 0.01, *** p < 0.001).

**Fig 6:**
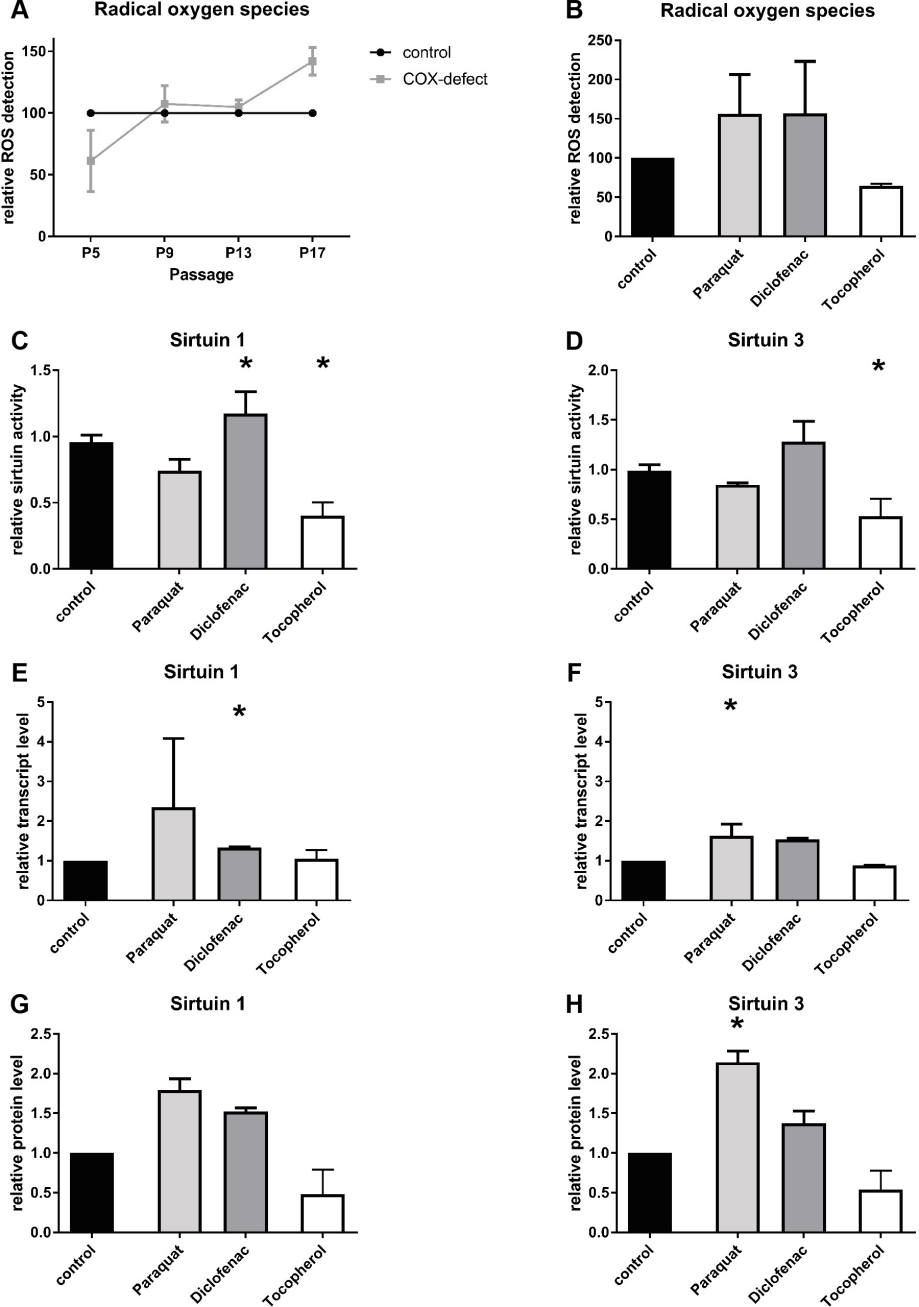
Effect of ROS modulation on sirtuin activity. (A) Relative amount of reactive oxygen species (ROS) in COX-deficient cells compared to control cells (B) Detection of relative amount of ROS in control cells treated with ROS inducers (paraquat, diclophenac) or ROS scavenger (tocopherol) (C-D) Enzymatic activities of SIRT1 (C) and SIRT 3 (D) in control cells treated with ROS inducers (paraquat, diclophenac) or ROS scavenger (tocopherol) (E-F) Relative transcript levels of SIRT1 (E) and SIRT 3 (F) after treatment with a ROS scavenger (tocopherol) or ROS inducers (paraquat, diclophenac) in control fibroblasts (G-H) Relative protein amount of SIRT1 (G) and SIRT 3 (H) in control cells treated with ROS inducers (paraquat, diclophenac) or ROS scavenger (tocopherol); (n = 3; all data are shown in mean ± SD; * p < 0.05).

The activity of complex IV in fibroblasts of a patient with residual COX-activity was induced after treatment with paeonol from 17 nmol/min per mg protein to 33 nmol/min per mg protein (Fig 5A, P1). In cells of a patient without residual activity (P2) it was not possible to induce complex IV activity by sirtuin induction. Due to a lack of further fibroblasts with residual complex IV activity we analysed the respiratory chain activities after pharmacological inhibition of sirtuins to show a direct link of modulating sirtuin activity and respiratory chain activity. SIRT1 and SIRT3 enzyme activity were significantly decreased by suramin treatment of control cells by 42% for SIRT1 and 45% for SIRT3 (Fig 5B). After tenovin-6 treatment SIRT1 was decreased in a non-significant manner by 13% and 20% for SIRT3 compared to DMSO treated controls. Our measurements of RC complex activities showed significant reductions of all complexes after treatment control cells with suramin. For complex I (Fig 5C) we measured a reduction with a mean of 23.9 nmol/min per mg protein, while complex II+III (Fig 5D) showed reduced enzymatic activity of 3.3 nmol/min per mg protein. The activity of complex IV (Fig 6E) was reduced by 58% to 19.3 nmol/min per mg protein and complex V activity (Fig 5F) decreased to 59.9 nmol/min per mg protein after suramin treatment. The enzymatic activities were lowered to a pathological level (complex I: 36 nmol/min per mg protein; complex II+III: 4 nmol/min per mg protein; complex IV: 20 nmol/min per mg protein; complex V: 71 nmol/min per mg protein) mimicking the state of mitochondriopathic patients. Treatment with tenovin resulted in a reduction in enzymatic activity of all examined complexes in a non-significant manner, except complex IV, where the reduction was also significant (p≤0.05). This reduction was less pronounced compared to suramin treatment. The treatment only reduced the activity of complex I and complex IV to a pathological level.

### Effect of ROS modulation on sirtuin activity

We measured ROS levels in COX-deficient fibroblasts compared to healthy fibroblasts. Surprisingly the ROS levels of COX-deficient cells were decreased by 40% in young cells. With aging of cells over different passages we observed an increase in ROS levels of patient cells compared to age-matched controls. In passage nine there was an increase of 7%, in passage 13 an increase of 5% and in passage 17 cells showed an increase of 42% compared to healthy fibroblasts (Fig 6A).

To further analyse the effect of ROS on sirtuins, we treated healthy fibroblasts with ROS inducers, paraquat and diclophenac, and the ROS scavenger tocopherol. As expected, the ROS inducers elevated the ROS levels by 56% in case of paraquat and 57% in case of diclophenac, while tocopherol reduced the ROS level by 36% (Fig 6B).

Effects of ROS modulation on maximal sirtuin activity were mostly similar to changes in protein amount. Tocopherol treatment resulted in a reduction to 40% of SIRT1 activity (Fig 6C) while it reduced SIRT3 activity by 47%. Diclophenac increased maximal SIRT1 activity by 25% and SIRT3 activity by 30%. Surprisingly paraquat treatment had a slightly decreasing effect on the maximal activities of SIRT1 (0.75 fold) and SIRT3 (0.85 fold) (Fig 6D). On transcript level, treatment with paraquat resulted in a 2.35 fold increased expression of *SIRT1* and a 1.62 fold increase of *SIRT3* expression. Treatment with diclophenac showed similar results with an increase of *SIRT1* expression by 34% and by 54% for *SIRT3* expression (Fig 6E and 6F). Interestingly, the ROS scavenger tocopherol had no effect on *SIRT1* expression and only a small effect on *SIRT3* with a reduction at transcript level by 13% (Fig 6E and 6F). In contrast to this, the relative protein levels were decreased for SIRT1 (0.48 fold) and SIRT3 (0.54 fold) after tocopherol treatment. Both ROS inducers showed similar results at protein levels compared to transcript levels. Paraquat increased SIRT1 protein amount by 79% and SIRT3 protein amount by 114%. The treatment with diclophenac was less effective. We measured an increase of 52% in case of SIRT1 and an increase of 37% for SIRT3 protein amount (Fig 6G and 6H).

## Discussion

Respiratory chain defects are serious, often life threatening multisystemic inborn errors of energy metabolism. Currently, only symptomatic treatment is available. Apart from energy deficiency, secondary phenomena like free radical species (ROS) production and NADH/FADH_2_ accumulation are of pathophysiological relevance. As energy metabolism is linked to sirtuin metabolism via NAD^+^ and sirtuins have regulatory functions at the level of respiratory chain complex activities we studied sirtuin activities in fibroblasts from COX-deficient patients.

We observed a significant reduction of mitochondrial sirtuin activities in skin fibroblasts from patients with COX-deficiency. Mitochondrial sirtuins are known to regulate RC function under physiological conditions [17,24] and are important regulatory elements of mitochondrial energy flux. Less sirtuins were produced at protein level, thus COX-deficiency had a modulating effect on mitochondrial as well as nuclear sirtuin content. Reduction of sirtuin activities in COX-deficiency has a negative impact on oxidative phosphorylation by compromising the already reduced energy flux across the RC even further. On the other hand, reduced activities of sirtuins result in a decreased energy flux by virtue of increased acetylation of respiratory chain complex subunits. This may be a protective mechanism in COX-deficiency as compromised electron flux will reduce formation of free radicals, hence oxidative stress. Down-regulation of NDUFA9 [24], a subunit of complex I at the entry point of RC, by reduced SIRT3-activity may be of particular importance in this context. On the other hand, ATPsynthase (complex V) works in reverse when RC-function is compromised, thus hydrolysing ATP [3]. Down-regulation of ATPsynthase by SIRT 3 inhibition [26] may contribute to conservation of cellular ATP in COX-deficiency. Furthermore, the regulation of ANT2 by SIRT4 may support this process [33].

The effect of COX-deficiency on sirtuins was independent of the cofactor NAD^+^ as in vitro assays were performed under cofactor saturation. Further analyses of our data showed that the reduction in sirtuin expression, protein level and maximal enzyme activity was less pronounced in fibroblasts of the two patients with residual complex IV activity. However, there was no link between alterations in NAD^+^-concentration and residual COX-activity.

Cellular NAD^+^ as an essential cofactor for sirtuin activity was reduced leading to further compromise of sirtuin-activities in the intact cell. This is probably due to a shift of the NADH/NAD^+^ -ratio towards NADH.

The mechanism leading to NAD^+^-independent sirtuin inhibition is not clear, ROS may be a regulatory element (secondary messenger) in this context. ROS levels in COX-deficient fibroblasts seem to be influenced by aging (higher passage numbers) being low in young patient cells and increased in aging cells (Fig 6A). The underlying mechanism needs to be addressed in further studies. All sirtuin measurements in patient cells were performed at low passage nubers and hence based on low ROS levels. Increasing ROS levels over time may be responsible for disease progression in patients. Some sirtuins are under the transcriptional control of FOXO transcription factors, which regulate the stress response [49,50]. Furthermore, sirtuins themselves regulate FOXO activity [30]. Schaar et al. pointed out that the subcellular localisation of ROS is also important for the function of ROS [51] which may also be relevant in sirtuin regulation. Modulating ROS by inducer and scavenger substances resulted in a sirtuin activation (ROS inducer) or sirtuin reduction (scavenger) (Fig 6). We could not detect any changes in transcript levels of *ampk*, *pparγ* and *pgc1α*. The slightly decreased protein level of AMPK may be secondary to an elevated AMP/ATP ratio in COX-deficiency. The lack of the competitive inhibitor ATP can result in an increased phosphorylation of AMPK and therefore in a higher kinase activity. This may be part of a mechanism to maintain a preferably intact mitochondrial signalling pathway via the AMPK/SIRT1/PGC1α axis as described by Canto *et*. *al*. [52].

COX-deficiency is a clinically and biochemically heterogeneous entity. In order to standardize COX-deficiency we incubated fibroblasts from healthy controls with cyanide. This led to inhibition of the oxygen consumption rate in a dose-dependent manner. Cyanide concentrations required for complete inhibition of oxygen consumption were relatively high when compared to concentrations needed to inhibit COX in isolated mitochondria [53]. This can be explained by the detoxification of cyanide via rhodanase in the intact cell [54].

When COX-deficiency was mimicked by cyanide incubation sirtuins were down-regulated at transcript, protein and activity level as observed in COX-deficient cells. Cell survival was not significantly affected in these experiments as judged by LDH-release and cell count (supplementary data S8 Fig and S1 Table). If the respiratory chain is blocked the NADH/NAD^+^-equilibrium should be shifted towards NADH, leading to reduced levels of NAD^+^ as observed in COX-deficient patient cells. However, NAD^+^-levels increased during cyanide incubation. The discrepancy between NAD+ concentration in patient cells and cyanide inhibited cells may be due to a difference between a chronic COX-defect in patient cells and a short-term treatment of seven days. Cells may switch to anaerobic glycolysis to maintain ATP supply resulting in higher NAD^+^ levels [55]. Additionally, it may be a secondary effect of ATP depletion. It is known that low ATP and high AMP levels induce AMPK activity which can result in an activation of NAMPT (nicotinamide phosphoribosyltransferase), the rate limiting enzyme of the NAD+-salvage pathway which is important for NAD^+^ generation [56,57].

In respiratory chain enzyme defects compensatory up-regulation of sirtuins might be expected in an attempt to activate residual enzyme activities. However, down-regulation of sirtuins was observed. Down-regulation of the respiratory chain enzymes is a physiological response to oxygen depletion in healthy cells (e.g. 3), inhibition of a respiratory chain complex has the same effect on the mitochondrial electrochemical gradient of the mitochondrial inner membrane as oxygen depletion. Thus, down-regulation of sirtuins is a physiological response to a block of electron flux across the respiratory chain.

A possible treatment option in mitochondrial disorders should aim at increasing deficient respiratory chain activities. Our results demonstrate that SRT1720 and paeonol are able to rescue intracellular sirtuin levels of COX-deficient patient cells in vitro. Additionally, we could show a rescue of complex IV activity in one COX-deficient patient cell line. A rescue in fibroblasts without residual COX-activity was not possible. Therefore, no further rescue experiments could be performed. As a proof of principle we inhibited sirtuin activity in healthy fibroblasts and analysed the enzymatic activity of different RC complexes showing that sirtuins can modulate COX-activity. A reduction of about 50% in SIRT1 and SIRT3 activity with suramin resulted in pathological activities of all RC complexes. Tenovin-6 inhibited sirtuin activity by 15–20% resulting in a less pronounced reduction of RC complex activities. Sirtuin activity seems to be essential for complete functionality of respiratory chain complexes [17,24–26,58]. Based on our findings, pharmacological modulation of sirtuins may be a promising option of patient treatment, as long as residual activity of the affected complex is present. Additional studies in animal models should be performed to investigate possible side effects of this modulation in vivo.

Furthermore, altered sirtuin levels may have secondary effects on metabolism. Beside a possible switch from glucose metabolism to lipid metabolism [7,59] other metabolic pathways, which are mediated by sirtuins are involved in growth processes and dysregulation, like altered TGF-β signalling. This may explain growth failure in patients with mitochondriopathies. Neuronal symptoms may also be a result of reduced sirtuin levels. A beneficial effect of high sirtuin levels in different brain tissues was described in the context of Alzheimer disease [60], Parkinson disease [61] and Huntington disease [62]. Furthermore, Kim *et*.*al*. [63] observed a beneficial effect of SIRT3 in a mouse model of excitotoxicity. SIRT 3 reduction may contribute to epilepsy in mitochondrial disorders in addition to ‘slow-onset excitotoxicity’.

Our study has limitations. We could not perform mutation analysis in COX-deficient patients as parents did not give informed consent or patients died and parents were lost to follow-up.Furthermore, fibroblasts are not an optimal tissue for assessing mitochondrial function as their energy demand is low. However, other tissue was not available for ethical reasons.

Further experimentation is needed to address pharmacological rescue.

In summary, we show that sirtuins are reduced in fibroblasts from patients with COX-deficiency or when COX was inhibited by cyanide in vitro. Activators of sirtuins may have a positive effect on residual COX-activity and hence on symptoms.

## Supporting information

S1 Fig Cellular respiration of cyanide treated cells measured with Seahorse XF. Treatment with increasing concentration of cyanide resulted in a decreased cellular respiration starting at a concentration of 2.5 mM NaCN and nearly represses respiration completely at a NaCN concentration of 7.5 mM.
(TIF)

S2 Fig Original uncropped Western Blots of SIRT1, SIRT3 and SIRT4 in Fig. These data files show the uncropped Odyssey FC generated fluorescence blot pictures of the bands used in the figures of the manuscript.
(TIF)

S3 Fig Original uncropped Western Blots of ACTB in Fig 1. These data files show the uncropped Odyssey FC generated fluorescence blot pictures of the bands used in the figures of the manuscript.
(TIF)

S4 Fig Original uncropped Western Blots of ACTB in Fig 2. These data files show the uncropped Odyssey FC generated fluorescence blot pictures of the bands used in the figures of the manuscript.
(TIF)

S5 Fig Original uncropped Western Blots of SIRT1, SIRT3 and SIRT4 Fig 2. These data files show the uncropped Odyssey FC generated fluorescence blot pictures of the bands used in the figures of the manuscript.
(TIF)

S6 Fig Original uncropped Western Blots of SIRT1, SIRT3 and SIRT4 in Fig 3. These data files show the uncropped Odyssey FC generated fluorescence blot pictures of the bands used in the figures of the manuscript.
(TIF)

S7 Fig Original uncropped Western Blots of ACTB in Fig 3. These data files show the uncropped Odyssey FC generated fluorescence blot pictures of the bands used in the figures of the manuscript.
(TIF)

S8 Fig LDH activity of cyanide treated Cells. The figure shows the LDH activity in control fibroblasts treated with increasing concentrations of NaCN (0 mM, 0.75 mM, 2.5 mM, 5 mM and 7.5 mM). The increasing NaCN concentrations do not result in a higher rate of cell death indicated by higher LDH activity.
(TIF)

S1 Table Cell count of NaCN treated cells. Fibroblasts treated with higher concentrations of NaCN (5 mM and 7.5 mM) showed decreased cell counts, indicating a reduced proliferation rate but did not differ in a statistical significant manner.
(TIF)
