# Supplementary figures and images for: Alterations of sirtuins in mitochondrial cytochrome c-oxidase deficiency
Source: PLoS One. 2017 Oct 23;12(10):e0186517. doi: 10.1371/journal.pone.0186517 (PMC5653369; doi:10.1371/journal.pone.0186517)

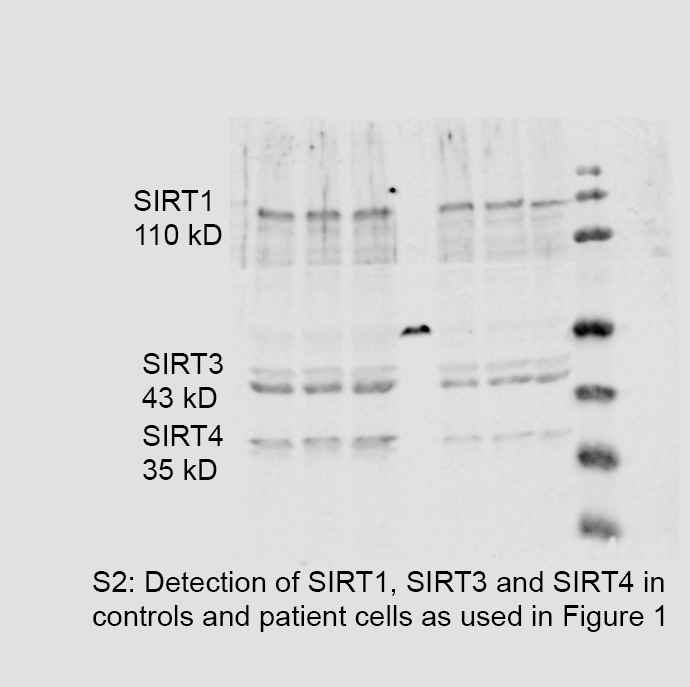

Supplement: S2 Fig — These data files show the uncropped Odyssey FC generated fluorescence blot pictures of the bands used in the figures of the manuscript. (TIF) [file pone.0186517.s002.tif]

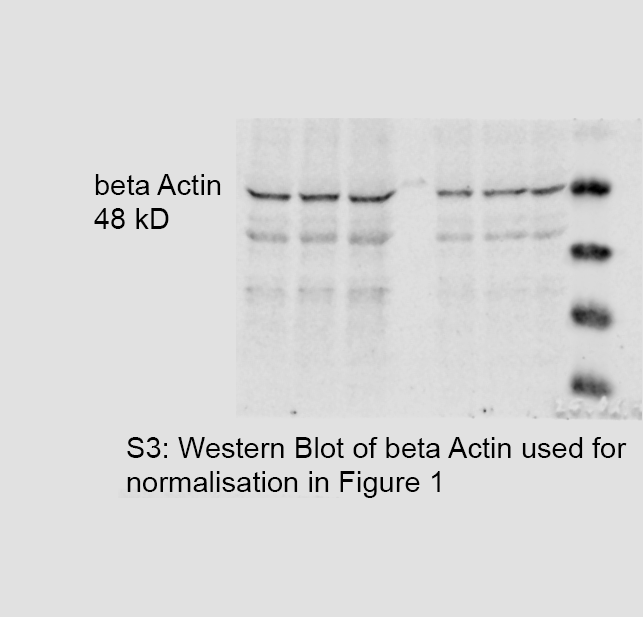

Supplement: S3 Fig — These data files show the uncropped Odyssey FC generated fluorescence blot pictures of the bands used in the figures of the manuscript. (TIF) [file pone.0186517.s003.tif]

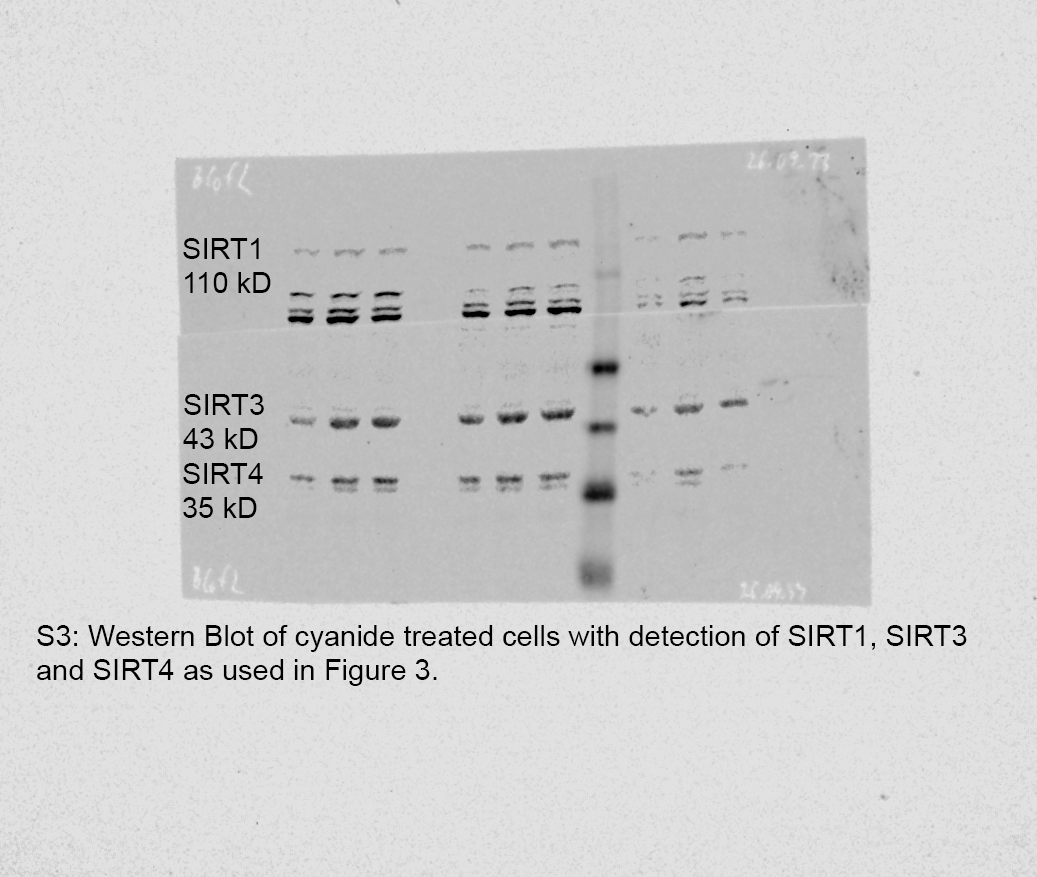

Supplement: S4 Fig — These data files show the uncropped Odyssey FC generated fluorescence blot pictures of the bands used in the figures of the manuscript. (TIF) [file pone.0186517.s004.tif]

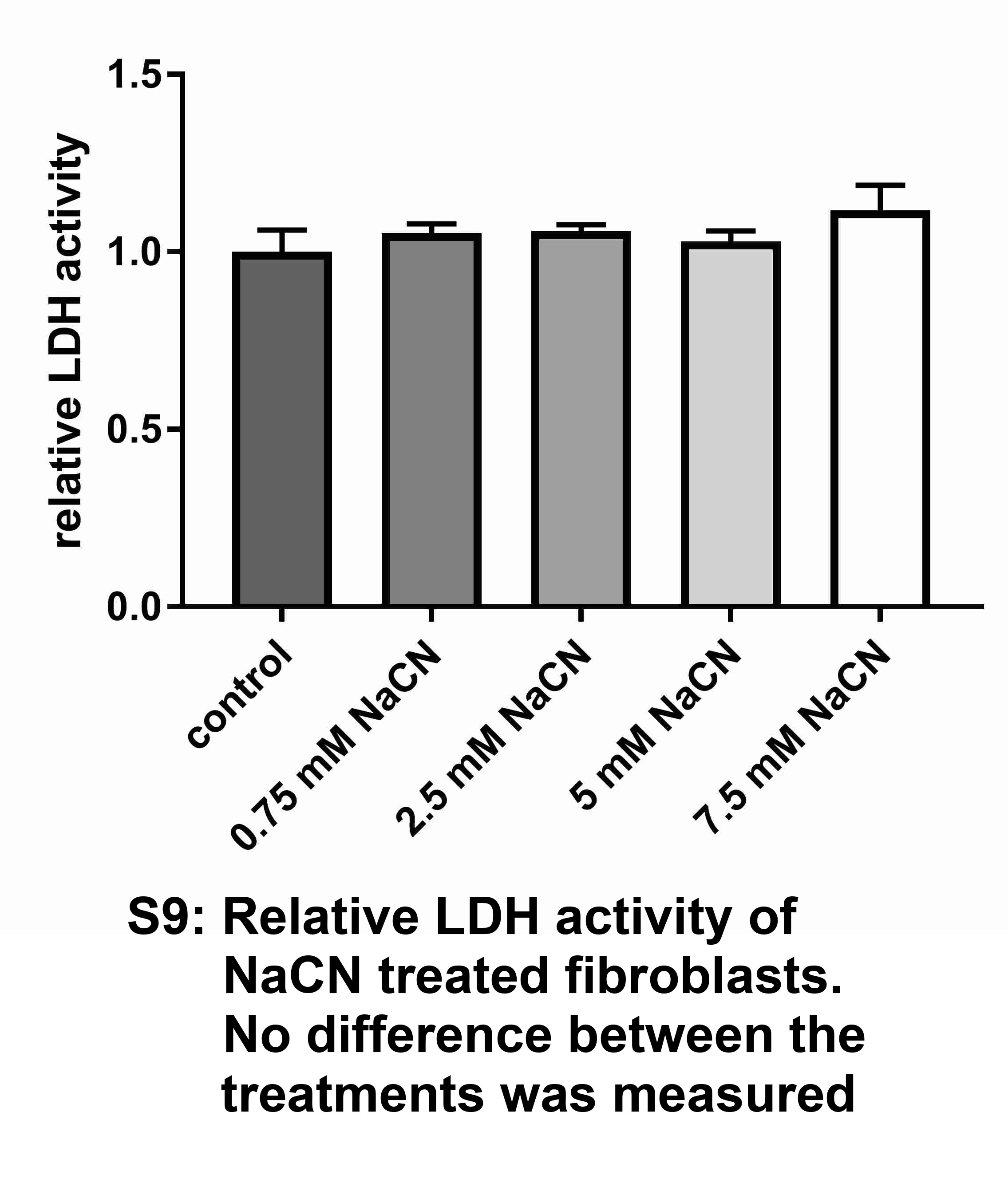

Supplement: S8 Fig — The figure shows the LDH activity in control fibroblasts treated with increasing concentrations of NaCN (0 mM, 0.75 mM, 2.5 mM, 5 mM and 7.5 mM). The increasing NaCN concentrations do not result in a higher rate of cell death indicated by higher LDH activity. (TIF) [file pone.0186517.s008.tif]

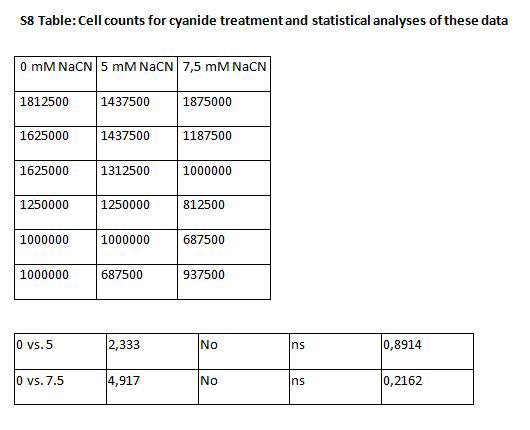

Supplement: S1 Table — Fibroblasts treated with higher concentrations of NaCN (5 mM and 7.5 mM) showed decreased cell counts, indicating a reduced proliferation rate but did not differ in a statistical significant manner. (TIF) [file pone.0186517.s009.tif]
